# Results Of *PMP22* Gene Deletion and Duplication Analysis in 100 Patients with Hereditary Neuropathy

**DOI:** 10.2478/bjmg-2026-0010

**Published:** 2026-10-14

**Authors:** SG Ergün, EF Perçin, MA Ergün

**Affiliations:** Hacettepe University Faculty of Medicine, Department of Medical Biology, Ankara, Turkiye; Gazi University Faculty of Medicine Department of Medical Genetics, Ankara, Turkiye

**Keywords:** *PMP22* gene, hereditary neuropathy, CMT1A, HNPP, PCR-RFLP

## Abstract

**BACKGROUND:**

Hereditary neuropathies, including CMT1A and HNPP, are commonly associated with *PMP22* gene dosage alterations. Although MLPA is widely used, cost-effective alternatives are still needed in resource-limited settings.

**METHODS:**

We analyzed *PMP22* gene deletions and duplications in 100 patients with suspected hereditary neuropathy using PCR-RFLP.

**RESULTS:**

*PMP22* deletions and duplications were detected in 18% and 8% of patients, respectively.

**CONCLUSIONS:**

Our findings support PCR-RFLP as a practical and accessible screening method for *PMP22* gene dosage abnormalities, particularly in settings where advanced molecular techniques are not readily available.

## INTRODUCTION

Hereditary neuropathies, including Charcot–Marie–Tooth disease (CMT; incidence ~1:2500) and hereditary neuropathy with liability to pressure palsies (HNPP; incidence ~1:6250), represent a clinically and genetically heterogeneous group of disorders affecting the peripheral nervous system [1,2]. Among these, CMT1A is the most common subtype and is primarily associated with duplication of the peripheral myelin protein 22 (*PMP22*) gene whereas deletion of the same gene underlies a substantial proportion of HNPP cases [3,4]. Proper *PMP22* gene dosage is essential for Schwann cell function and peripheral nerve myelination, and its dysregulation leads to characteristic neuropathic phenotypes [ 5, 6, 7].

Accurate detection of *PMP22* gene dosage alterations is therefore critical for definitive diagnosis, patient management, and genetic counseling. Multiplex ligation-dependent probe amplification (MLPA) is widely accepted as the gold standard method due to its high sensitivity and reliability [1,2]. However, access to MLPA may be limited in certain clinical settings due to cost and technical requirements.

In such contexts, simpler polymerase chain reaction (PCR)-based approaches may offer a practical alternative for initial screening. In this study, we evaluated the applicability of PCR-restriction fragment length polymorphism (RFLP) for detecting *PMP22* gene deletions and duplications in patients with suspected hereditary neuropathy. Using samples obtained from routine clinical practice, we aimed to assess whether this method could serve as a cost-effective and accessible option for molecular diagnosis in settings where advanced techniques are not readily available.

## MATERIALS AND METHODS

### Study Population

This study represents a retrospective evaluation of routine diagnostic genetic testing and included 100 patients referred to the Medical Genetics Laboratory between 2010 and 2013 for genetic testing due to clinical findings suggestive of hereditary neuropathy. Patients were evaluated by neurologists based on neurological examinations, family history, and nerve conduction studies; however, detailed clinical and electrophysiological data were not consistently available for all patients. Patients with insufficient DNA quality or incomplete clinical data were excluded from the analysis. This study was conducted in accordance with the Declaration of Helsinki. All genetic analyses were performed after obtaining informed consent from patients.

### Molecular Studies

In this retrospective study, 100 patients with a preliminary diagnosis of hereditary neuropathy were included. Peripheral venous blood samples (5 mL) were collected in EDTA-containing tubes. Genomic DNA was isolated using a commercially available kit (Macherey-Nagel, Germany) according to the manufacturer’s instructions.

*PMP22* gene deletion and duplication analyses were performed using the PCR-RFLP method with the following primers [1,3]:

Forward: 5′-AGGTTGTTTACTCCTTCTTC-3′
CMT1A Reverse: 5′-AGATGGAATAGTAGAGCTCAC-3′
HNPP Reverse: 5′-AGATGGAATAGTAGAGTGAG-3′

Long-range PCR amplification was performed using the Expand Long Template PCR System (Roche, Germany) according to the manufacturer’s instructions, in a total reaction volume of 50 µL. The thermal cycling conditions consisted of an initial denaturation at 94°C for 3 minutes, followed by 10 cycles of 94°C for 30 seconds, 54°C for 30 seconds, and 68°C for 3 minutes, then 25 cycles of 94°C for 30 seconds, 54°C for 30 seconds, and 68°C for 3 minutes, with a final extension at 68°C for 7 minutes.

PCR amplification yielded a 3637 bp product. The amplified products were digested with 10 U EcoRI (NEB, R0101S, USA) in 1× NEBuffer at 37°C according to the manufacturer’s instructions and subsequently separated on a 1% agarose gel at 120 V for 2 hours. Complete digestion was confirmed by including control samples (undigested and uncut controls) and verifying the expected fragment patterns.

Following EcoRI enzyme digestion, heterozygous samples from CMT1A cases showed two fragments at 3293 and 344 bp, indicating gene duplication whereas no digestion was observed in wild-type samples (3637 bp). In HNPP cases, wild-type samples produced 3293 bp and 344 bp fragments after digestion while samples with *PMP22* deletion produced a 3637 bp fragment after digestion (Figure 1).

**Figure 1:**
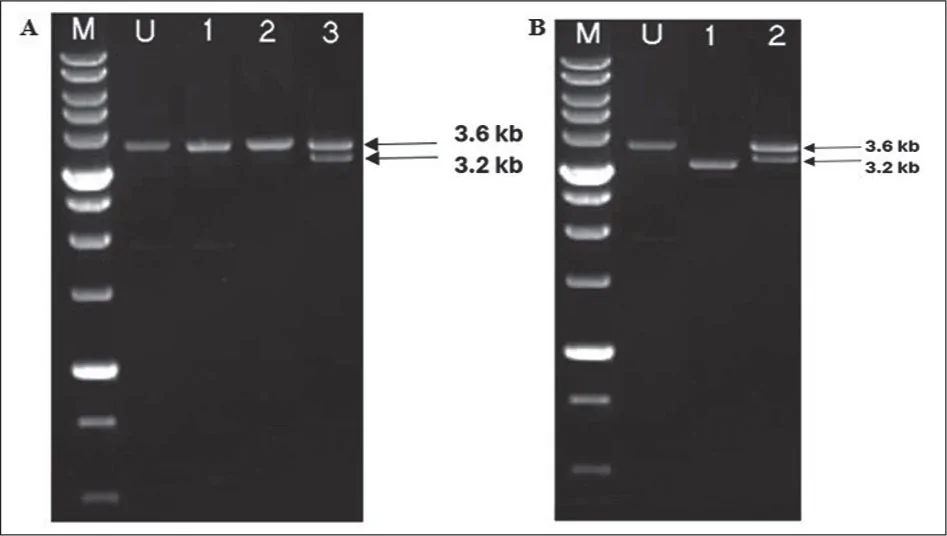
PCR-RFLP image of *PMP22* gene analysis for both CMT1A and HNPP. **A)** M: DNA marker (1 kb), U: Uncut, 1,2:wild type 3:heterozygous *PMP22* duplication (CMT1A). **B)** M: DNA marker (1 kb), U: Uncut, 1:wild type, 2:heterozygous *PMP22* deletion (HNPP).

## RESULTS

A total of 100 patients were included in the study, comprising 52 males (52%) and 48 females (48%). The mean age of the cohort was 46.6 ± 13.1 years. Based on clinical evaluation before genetic testing, 52 patients (52%) were referred with a suspicion of CMT. In comparison, 48 patients (48%) were suspected of HNPP. The demographic and clinical characteristics of the study population are summarized in Table 1.

**Table 1.** Demographic and clinical characteristics of the study cohort.

**Variable**	**Value**
Total patients (n)	100
Male, n (%)	52 (52%)
Female, n (%)	48 (48%)
Age (years, mean ± SD)	46.6 ± 13.1
Suspected CMT, n (%)	52 (52%)
Suspected HNPP, n (%)	48 (48%)

Among the 100 patients analyzed by PCR-RFLP, *PMP22* gene deletion was detected in 18 patients (18%), consistent with HNPP while duplication was identified in 8 patients (8%), consistent with CMT1A. No *PMP22* gene dosage alteration was detected in the remaining 74 patients (74%). All samples were successfully analyzed, and no technical failures were observed. The distribution of *PMP22* gene alterations according to clinical suspicion is summarized in Table 2.*PMP22* gene abnormalities were predominantly detected in accordance with the clinical pre-diagnosis, with deletions mainly observed in patients suspected of HNPP and duplications in those suspected of CMT. However, due to the retrospective design and limited availability of detailed clinical and electrophysiological data, a comprehensive genotype–phenotype correlation analysis was not possible.

**Table 2.** Distribution of *PMP22* gene alterations according to clinical suspicion.

**Clinical suspicion**	***PMP22* deletion (HNPP)**	***PMP22* duplication (CMT1A)**	**No alteration**	**Total**
CMT (n=52)	-	8	44	52
HNPP (n=48)	18	-	30	48
Total	18	8	74	100

## DISCUSSION

In the present study, *PMP22* gene dosage alterations were identified in 26% of patients with suspected hereditary neuropathy, including deletions in 18% and duplications in 8%. These findings were consistent with previous studies demonstrating that *PMP22* gene abnormalities represent a substantial proportion of hereditary neuropathies. The relatively higher frequency of deletions observed in our cohort may be explained by referral patterns as patients with clinical features suggestive of HNPP are more likely to undergo targeted genetic testing.

Hereditary neuropathies represent a clinically and genetically heterogeneous group of disorders affecting the peripheral nervous system, with a wide spectrum of clinical presentations and underlying genetic causes [8]. Alterations in the *PMP22* gene dosage, including deletions and duplications, are among the most common molecular mechanisms underlying these conditions [9]. Patients carrying PMP22 pathogenic variations may present with variable disease onset ranging from early childhood to late adulthood, reflecting the complex genotype–phenotype relationship [10].

The relationship between *PMP22* gene dosage and clinical phenotype remains complex. In patients with *PMP22* deletions, genotype–phenotype correlations may vary, resulting in varying degrees of nerve dysfunction. Similarly, the mechanisms underlying HNPP and CMT1A are not fully understood, and factors such as gene dosage effects and genomic context have been suggested to influence disease severity [4,12]. These findings highlight the importance of accurate molecular diagnosis not only for confirmation but also for clinical interpretation and patient management.

Various molecular techniques, including fluorescence *in situ* hybridization (FISH), Southern blot, pulsed-field gel electrophoresis (PFGE), PCR-RFLP, and MLPA, have been developed to detect *PMP22* gene deletions and duplications [1,2]. Among these, MLPA is widely accepted as the gold standard due to its high sensitivity and reliability [1,8]. However, previous comparative studies have demonstrated that while PCR-RFLP can detect *PMP22* gene deletions and duplications, it has lower sensitivity and accuracy than MLPA [1]. Nevertheless, PCR-RFLP remains a practical and cost-effective alternative that can be applied as a first-line screening tool, particularly in settings where access to advanced molecular techniques is limited. Our findings support the applicability of PCR-RFLP in routine diagnostic workflows for distinguishing between deletion, duplication, and wild-type patterns.

Importantly, no *PMP22* gene dosage alteration was detected in 74% of the patients, highlighting the marked genetic heterogeneity of hereditary neuropathies. This observation suggests that pathogenic variants in other genes may contribute to the disease in a substantial proportion of cases, underscoring the need for more comprehensive genetic testing in patients with negative *PMP22* results [8].

This study has several limitations. First, due to the retrospective design, detailed clinical and electrophysiological data were not consistently available, limiting comprehensive genotype–phenotype correlation analysis. Second, systematic familial testing was not performed or recorded for all patients, which restricts further confirmation of inheritance patterns. Third, clinical severity data were not uniformly available, preventing classification of patients into mild, moderate, and severe categories. Finally, PCR-RFLP may not detect all types of genomic rearrangements, and the lack of validation with MLPA represents an additional limitation.

In conclusion, our results demonstrate that PCRRFLP is a useful, accessible, and cost-effective method for detecting *PMP22* gene deletions and duplications in a routine diagnostic setting. While it does not replace advanced techniques such as MLPA, it can serve as an effective first-line screening tool, particularly in resource-limited clinical environments. Accurate molecular diagnosis remains essential for genetic counseling, family planning, and clinical management of patients with hereditary neuropathies.

## CONCLUSIONS

In conclusion, our findings demonstrate that PCRRFLP is a practical and cost-effective method for detecting *PMP22* gene deletions and duplications in patients with suspected hereditary neuropathy. Although it does not replace advanced techniques such as MLPA, it may serve as a reliable first-line screening tool, particularly in resource-limited settings. Furthermore, accurate detection of *PMP22* gene alterations is crucial for clinical diagnosis and supports appropriate genetic counseling and patient management.
